# A systematic review of personality and musculoskeletal disorders: evidence from general population studies

**DOI:** 10.3389/fpsyt.2024.1288874

**Published:** 2024-05-21

**Authors:** Shae E. Quirk, Heli Koivumaa-Honkanen, Risto J. Honkanen, Mohammadreza Mohebbi, Amanda L. Stuart, Jeremi Heikkinen, Lana J. Williams

**Affiliations:** ^1^ Institute for Mental and Physical Health and Clinical Translation, School of Medicine, Deakin University, Geelong, VIC, Australia; ^2^ Institute of Clinical Medicine, Psychiatry, University of Eastern Finland, Kuopio, Finland; ^3^ Kuopio Musculoskeletal Research Unit (KMRU), Institute of Clinical Medicine, University of Eastern Finland, Kuopio, Finland; ^4^ Biostatistics Unit, Faculty of Health, Deakin University, Geelong, VIC, Australia; ^5^ Barwon Health, University Hospital Geelong, Geelong, VIC, Australia

**Keywords:** personality disorder, PD, musculoskeletal disorders, musculoskeletal diseases, review, systematic review, comorbidity

## Abstract

**Introduction:**

We conducted a systematic review to evaluate the quality and extent of evidence on associations between personality disorders (PDs) and musculoskeletal disorders (MSDs) in population-based studies, since these disorders are leading causes of disease burden worldwide.

**Methods:**

A search strategy of published, peer-reviewed and gray literature was developed in consultation with a liaison librarian and implemented for Embase, CINAHL Complete, Medline Complete, and PsycINFO via the EBSCOhost platform from 1990 to the present and CORDIS and ProQuest Dissertations & Theses Global, respectively. The inclusion criteria were as follows: I) general population participants aged ≥15 years; II) self-report, probable PD based on positive screen, or threshold PD according to the DSM-IV/5 (groupings: any, Clusters A/B/C, specific PD) or ICD-10/11; III) MSDs identified by self-report or ICD criteria (arthritis, back/neck conditions, fibromyalgia, osteopenia/osteoporosis) and III) cohort, case-control, and cross-sectional study designs. Two reviewers independently screened articles and extracted the data. Critical appraisal was undertaken using the Joanna Briggs Institute checklists for systematic reviews of etiology and risk. A descriptive synthesis presents the characteristics of included studies, critical appraisal results, and descriptions of the main findings. This review adhered to the PRISMA (Preferred Reporting Items for Systematic Reviews and Meta-Analyses) guidelines.

**Results:**

There were 11 peer-reviewed, published articles included in this review (n = 9 cross-sectional and n = 2 case-control studies); participants were ≥18 years in these studies. No published gray literature was identified. Semi-structured interviews were the most common method to ascertain PDs; all studies utilized self-reported measures to identify MSDs. Overall, we detected limited and conflicting evidence for associations between PDs and MSDs.

**Discussion:**

The main result may be explained by lack of population-based longitudinal evidence, heterogenous groupings of PD, and few comparable cross-sectional and case-control studies. Strengths of the review include a comprehensive search strategy and a discussion of mechanisms underlying possible associations between PDs and MSDs.

**Conclusions:**

The quality of most studies included in this review that examined associations between PD and MSDs in general population adults was high. However, the results demonstrated limited and conflicting evidence for these associations, in part, due to lack of comparable evidence, which should be addressed in future research.

**Systematic review registration:**

https://www.crd.york.ac.uk/prospero/, identifier CRD42021243094.

## 1 Introduction

Mental disorders and musculoskeletal disorders (MSDs) are each leading contributors to years lived with disability (YLD) (1). Personality disorders (PDs) are a common form of mental disorders, with a worldwide prevalence estimate of approximately 8% (2). PDs manifest as difficulties with emotion regulation, interpersonal relating, and adaptive functioning such as coping with daily life stressors and demands (3, 4). Existing classifications characterize PD as 10 distinct categorical disorders that are organized into Clusters A (“odd-eccentric” features), B (“dramatic/emotional/erratic” features), and C (“anxious/fearful” features) (5). Contemporary approaches to the classification of PD have resulted in the International Classification of Diseases (ICD-11) adopting a dimensional approach to classification focusing on patterns of traits that contribute to impairment in global personality functioning (6). Separately, MSDs are a group of conditions or consequences from injury that affect bones (i.e., osteoporosis, osteopenia), joints (i.e., types of arthritis such as osteoarthritis, rheumatoid arthritis, and psoriatic arthritis), muscles, and other soft tissues, as well as those that implicate multiple body areas or systems (i.e., chronic back/neck pain and fibromyalgia) (7). These MSDs can result in courses of acute or chronic painful symptoms, significantly restricting mobility and functioning, and leading to increased morbidity and mortality [see literature review by Briggs et al. (8)]. There is growing evidence of high occurrences of comorbid PD with common types of MSDs in clinical populations (9). However, awareness of these associations in the general population is still limited.

Among patients with chronic back/neck pain, the proportion of PD is reported to range between 43.6% and 69.6% (clinically based studies) (10–12). Longitudinally, the occurrence of chronic back pain in patients with remitted and non-remitted borderline PD is 47.8% and 57.7%, respectively (at 16 years of follow-up) (13). In addition, PD is common among patients with fibromyalgia with frequencies ranging from 8.7% to 65.0% in clinical studies (14–18). Moreover, in a retrospective cohort study using hospitalization/physician claim data, patients with rheumatoid arthritis had increased incidence of PD (incident rate ratio = 1.61; 95% CI: 1.29–1.99) compared to controls (matched 5:1 on sex, age, and region of residence in Manitoba, Canada) as well as other types of immune-mediated inflammatory diseases (19). Moreover, there is longitudinal evidence that remission status among patients with borderline PD is associated with osteoarthritis over the long term with 4.0% (remitted) and 15.5% (non-remitted) having osteoarthritis at the study baseline and 11.9% (remitted) and 26.8% (non-remitted) after 16 years (13, 20). However there was no population-based control group. There is also clinical cross-sectional evidence to suggest patients (women only) with borderline PD may have reduced bone mineral density (BMD) and be at risk of osteoporosis (21, 22).

Improved recognition of these associations in a population-based setting is warranted; evidence from a large epidemiological study showed that as separate categories of disorders, PDs and MSDs, such as arthritis, are associated with significant population-level quality-adjusted life year (QALY) losses, which suggests a high overall burden to individuals and society (23).

Recently, we undertook a scoping review of the comorbidity of PD and MSDs, which included existing reviews (9). It revealed that there were no existing systematic reviews that incorporated critical appraisal of the evidence, or meta-analyses, and none that focused on population-based associations between PD and MSDs. Therefore, we developed and published a protocol, which outlines the methodological approach to conduct a systematic review, and address this gap in the literature (24). Prior to commencing the conduct, we confirmed no existing systematic review on this topic via a search of PROSPERO, Open Registries, and Medline Complete, CINAHL Complete, and PsycInfo databases (EbscoHost platform). The systematic review is needed to assess the quality of existing evidence and quantify associations to provide directions for future research and practice. In addition, evaluating the evidence will enable inferences to be made about these associations in the population, along with potential associated needs (23).

Therefore, a systematic review was undertaken, with the objective of evaluating population-based epidemiological associations between PD and burdensome MSDs including arthritis, back/neck pain, fibromyalgia/muscular pain, and osteopenia/osteoporosis. These MSDs were selected as outcomes for this review, as we recently scoped the evidence on this topic (9) and identified these MSDs as possibly highly comorbid with PD in clinical and/or general population settings. In addition, we identified emerging evidence of associations with poorer bone health (25). In accordance, the research questions were as follows:

1. Is there an association between PDs and arthritis, back/neck pain, fibromyalgia/muscular pain, and osteopenia/osteoporosis and/or “any” of these conditions?
1.1. What methodological characteristics explain the heterogeneity in results?
2. For the question above, what is the quality and levels of evidence for these associations?

## 2 Methods

The protocol for this review is published (24), registered with PROPSERO (CRD42021243094), and was conducted in accordance with the Preferred Reporting Items for Systematic review and Meta-Analysis (PRISMA) (26). It was also guided by the Joanna Briggs Institute resources for conducting systematic reviews of etiology and risk (27).

### 2.1 Inclusion criteria

The Population, Exposure, Outcome (PEO) framework (27) was used to characterize the inclusion and exclusion criteria for this review and is presented in **Table 1**. Eligible study designs were population based, observational cross-sectional (analytical), case-control, or cohort studies. Therefore, intervention, qualitative, and descriptive study designs were not considered eligible. No restrictions on participant characteristics or language of publications were applied.

**Table 1:** Inclusion and exclusion criteria according to the Population, Exposure, Outcome (PEO) framework.

PEO framework	Inclusion(s)	Exclusion(s)
***Population***	General population participants aged 15 years and over • No restrictions on characteristics • No restrictions on language	• Participants under the age of 15 years • Studies conducted in clinical settings
***Exposure(s)***	PDs according to existing classifications: • DSM-IV/5 • ICD-10 or ICD-11 criteria Assessed/identified by: • Structured/semi- structured interview administered by a trained interviewer (i.e., graduate with a relevant qualification or lay interviewer) or expert (i.e., relevant health professional) • Screening/self- reported instruments	• Does not examine PDs according to the inclusion criteria
***Outcome(s)***	Presence of (yes/no) • Arthritis • Back/neck pain • Fibromyalgia/ muscular pain • Osteopenia/ osteoporosis • Any of these conditions Assessed/identified by: • ICD criteria, diagnosed by a relevant health professional, or other relevant clinical criteria reported in linked medical records (i.e., “expert diagnosis”) • Self-reported from questionnaire responses or semi-structured interviews (i.e., “self-report”)	• Does not examine MSDs according to the inclusion criteria • MSDs that are grouped with other conditions • Non-MSD-related pain conditions/types of chronic pain such as cancer-related pain, chronic fatigue syndrome, headache, inflammatory bowel disease, migraine, temporomandibular joint dysfunction •Does not examine MSDs according to the inclusion criteria

### 2.2 Evidence sources

Peer-reviewed and published gray literature evidence sources were restricted to those published in or after 1990. For this review, published gray literature included dissertations published in ProQuest Dissertations & Theses Global or reports/publications deriving from relevant projects/programs housed in the European Commission’s Community Research and Development Information Service (CORDIS). These evidence sources were considered due to the potential to extract relevant epidemiological data. Additional information sources were identified by screening and reviewing the reference lists of eligible studies. Further details are presented below (see section on search strategy).

### 2.3 Study identification and selection

#### 2.3.1 Search strategy

A comprehensive search strategy was developed to identify eligible evidence sources. First, in consultation with a liaison librarian (LG), a pilot search was developed and implemented in Medline Complete using the EBSCOhost platform on 26 August 2021 as part of the protocol development (24). Text words contained in the titles and abstracts of relevant articles were identified, and the search was developed using index terms (MeSH) and keywords, Boolean operators, truncations, and explode functions. A “gold set” list (25, 28–34) of relevant articles were identified from a recent scoping review (9) and used to test the preliminary search. All gold set articles were returned in the pilot search yield. Next, the search for Medline Complete was translated for CINAHL Complete and APA PsycINFO (via EBSCOhost), and Embase. Gray literature was searched using an adapted search for ProQuest Dissertations & Theses Global databases and CORDIS. The complete search was implemented on 16 September 2021 and updated on 20 February 2024 (see **Supplementary Tables 1**-**6**). Finally, the reference lists of included studies were exported using SCOPUS.

**Figure 1:**
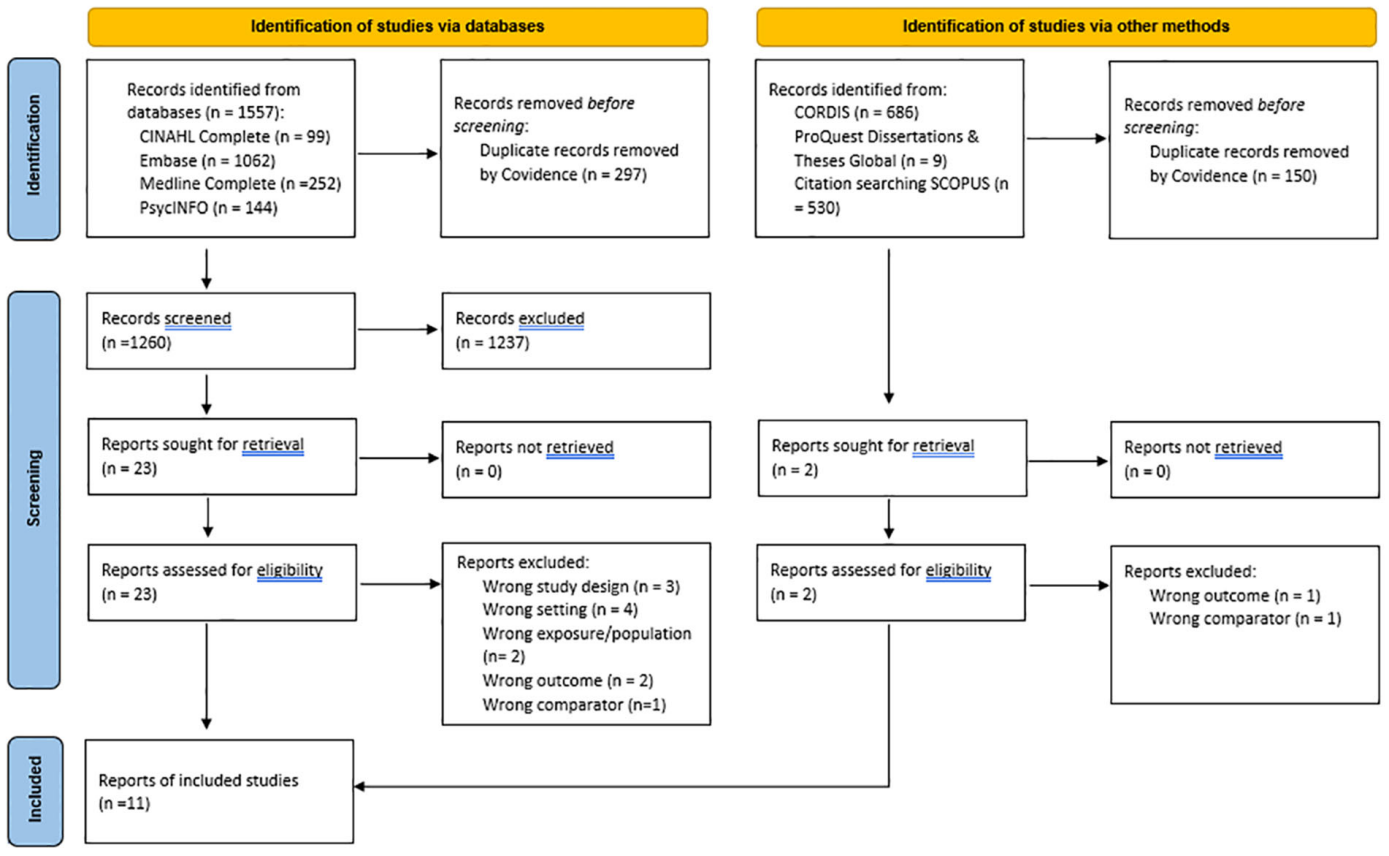
PRISMA 2020 flow diagram adapted from “Page MJ, McKenzie JE, Bossuyt PM, Boutron I, Hoffmann TC, Mulrow CD, et al. The PRISMA 2020 statement: an updated guideline for reporting systematic reviews. BMJ 2021;372:n71. doi: 10.1136/bmj.n71.

Assisted by the liaison librarian (LG), one reviewer (SEQ) implemented the search strategy and managed the records. The records from the combined searches were exported to Mendeley reference management software and Covidence (35) with duplicates removed. The flow of citations and reasons for exclusion at the full-text review are presented in **Figure 1** and in the results (see Section 3).

#### 2.3.2 Search strategy

Two reviewers (LJW and SEQ) conducted pilot testing of the inclusion and exclusion on a sample of 25 randomly selected articles from the search yield prior to screening. Next, the same two reviewers independently undertook screening of titles/abstracts and review full-text review of articles in Covidence. Discrepancies at the screening and full-text review phase were resolved in one consensus meeting each. One reviewer (SEQ) screened the records identified from the other methods. The final list of published peer-reviewed and gray literature was confirmed by the supervising author (LJW).

### 2.4 Data collection, extraction, and reporting

#### 2.4.1 Critical appraisal of individual studies

Two reviewers (ALS and SEQ) independently assessed for risk of bias of the included studies using an adapted version of the critical appraisals tools by the Joanna Briggs Institute (36). Disagreements were resolved between the reviewers in one consensus meeting.

The critical appraisal tools assess pertinent biases in observational studies, including potential selection bias, information bias, and confounding, according to the relevant study design (36). Specifically, for the cross-sectional studies, we rated seven items concerning a) the inclusion/exclusion criteria, b) the description of the sample/representativeness, c) reliability and validity of the exposure(s), d) whether confounding factors were identified and measured, e) whether confounding factors were accounted for in study design/analysis, f) reliability and validity of the outcome(s), and g) appropriateness of statistical analyses. For the case-control studies identified, nine items were rated regarding a) comparison group representativeness (of source population), b) appropriateness of recruitment/matching of cases and controls, c) appropriateness of criteria to identify cases and controls, d) reliability and validity of the exposure(s), e) measurement of exposure(s) for cases and controls, f) whether confounding factors were identified and measured, g) whether confounding factors were accounted for in study design/analysis, h) reliability and validity of the outcome(s), and i) appropriateness of statistical analyses (36). The items were rated as follows: 0) did not satisfy the criteria (i.e., no) and 1) satisfied the criteria (i.e., yes). We summed the ratings to derive a critical appraisal score for each study (0–7 for cross-sectional studies; 0–9 for case-control studies.) This method was similarly applied in a separate published review on the prevalence of PDs in the community (2). The results of the critical appraisal are presented in **Table 2**.

**Table 2:** Characteristics of included studies.

Citation and country	Study and population characteristics			PD assessment		MSD assessment		Critical appraisal score
	Study design (data collection/follow-up period)	Study population; sample size (*n*)	Mean age (SD)/median (IQR)/age range Sex: % female	PD	Assessment • Classification • Tool • Administration	MSD	Assessment • Tool • Administration	
El-Gabalawy et al. (29) USA	Cross-sectional (NESARC Wave 2; 2004-2005)	Wave 2 NESARC participants N: 34,653	Aged 18+ Sex: 52.1%	• Borderline PD	• DSM-IV • AUDADIS-IV • Lay interviewer	Arthritis	• AUDADIS-IV • Self-reported	7/8
Goldstein et al. (30) USA	Cross-sectional (NESARC Wave 1; 2001–2002)	Wave 1 NESARC participants N: 43,093	48 (13.3) Sex: nr	• Antisocial PD	• DSM-IV • AUDADIS-IV • Lay interviewer	Arthritis	• AUDADIS-IV • Self-reported	6/8
McWilliams et al. (31) USA	Cross-sectional (NESARC Wave 1; 2001–2002)	Wave 1 NESARC participants N: 43,093	Aged 18+ Sex: nr	• Paranoid, schizoid, histrionic, antisocial, avoidant, dependent, obsessive–compulsive PDs	• DSM-IV • AUDADIS-IV • Lay interviewer	Arthritis	• AUDADIS-IV • Self-reported	7/8
McWilliams & Higgins (28), USA	Cross-sectional (NCS Part II; 2001–2003)	Community-based respondents enrolled in Part II of the NCS-R N: 5,692	Aged 18+ Sex: nr	• Borderline PD criteria mean scores	• ICD-10 • IPDE screener using borderline PD items • Self-report	• Arthritis • Chronic spinal pain	• Questionnaire • Self-reported	6/8
Olssøn & Dahl (37), Norway	Case-control (HUBRO; May 2000–September 2001)	Community-based respondents to the HUBRO study health Survey N: 2,214 Cases: 369 Controls: 1,845	Aged 30+ Sex: 48%	• PD-positive screen^*^	• DSM-IV • 5 items from the IPDS • Method nr	• Fibromyalgia • Muscular pain	• Questionnaire • Self-reported	9/10
Olssøn & Dahl (34), Norway	Case-control (HUBRO; May 2000–September 2001)	Community-based respondents to the HUBRO study health Survey N: 280 (cases) N: 1,400 (controls)	Aged 30+ Sex: 65%	• Avoidant PD-positive screen^†^	• DSM-IV • Avoidant PD items of IPDS • Method nr	Muscular pain (affecting work ability)	• Questionnaire • Self-reported	4/10
Powers & Oltmanns (32), USA	Cross-sectional (SPAN; dates nr)	Community-based residents aged 55–64 years enrolled in the SPAN N: 1,051	59.4 (2.7) Sex: 53%	• Borderline PD	• DSM-IV • SIDP-IV • Trained interviewer (clinician reported); informant-reported, self-reported	Arthritis	• Health section of the DIS • Self-reported	6/8
Quirk et al. (38) USA	Cross-sectional (NESARC pooled; Wave 1 2001–2002 and Wave 2 2004–2005)	Wave I and 2 NESARC participants N: 34,653	Aged 20+ Sex: 52.1%	• Any PD • Clusters A, B, and C PDs • Paranoid, schizoid, schizotypal, antisocial, borderline, histrionic, narcissistic, avoidant, dependent, obsessive–compulsive PDs	• DSM-IV • AUDADIS-IV • Lay interviewer	Arthritis	• AUDADIS-IV • Self-reported	7/8
Quirk et al. (33) Australia	Cross-sectional (GOS; 2011–2014)	Community-based women enrolled in the GOS in southeastern Australia N: 765	56.8 (42.7–68.9) Sex: 100%	• Any PD • Cluster A, B & C PDs	• DSM-IV • SCID-II • Trained interviewer	Arthritis (grouped): osteoarthritis, ankylosing spondylitis, psoriatic arthritis, rheumatoid arthritis, or treated gout (medications used for gout or hyperuricemia)	• Questionnaire • Self-reported and/or medication use history	6/8
Sleurs et al. (39) USA	Cross-sectional (NESARC Wave 3 (2012-2013)	Wave III NESARC participants N: 36,309	18+ Sex: 51.16% (no fibromyalgia); Sex: 87.48% (fibromyalgia present)	• Schizotypal, borderline, and antisocial PDs	• DSM-5 • AUDADIS-5 interviewer	Fibromyalgia	• AUDADIS-5 • Self-reported	6/8
Williams et al. (25) Australia	Cross-sectional (GOS; 2011–2014)	Community-based women enrolled in the GOS in southeastern Australia N: 696	56.8 (42.7–68.9) Sex: 100%	• Any PD • Clusters A, B, and C PDs	• DSM-IV • SCID-II • Trained interviewer	• Osteoporosis • BMD	• Dual-energy X-ray absorptiometry • Areal BMD (g/cm^2^) measured at the posterior–anterior spine, femoral neck (hip), and total body including head • Osteoporosis (yes/no) defined as BMD T-score of <-2.5 at the spine and/or hip	6/8

AUDADIS, Alcohol Use Disorder and Associated Disabilities Interview Schedule; BMD, bone mineral density; Critical appraisal score, undertaken using the Joanna Briggs Institute checklists for systematic reviews of etiology and risk; DIS, Diagnostic Interview Schedule; DSM, Diagnostic and Statistical Manual of Mental Disorders, 4th Edition; ICD, International Classification of Diseases; IPDE, International Personality Disorder Examination; IPDS, Iowa Personality Disorder Screen; SCID-II, Structured Clinical Interview for DSM Axis II Disorders; SIDP-IV, Structured Interview for DSM-IV Personality; nr, not reported.*Cut-off ≥6. †Endorsement on two avoidant PD items of the Iowa Personality Disorder Screen plus the Social Phobia Inventory (short version) for generalized social anxiety disorder (sum score ≥6).

#### 2.4.2 Data extraction

A data extraction form was developed in consultation with a statistician (MM) using Microsoft Excel. One reviewer (SEQ) extracted the data, and another (ALS) validated the data with discrepancies resolved in one meeting. The primary outcome(s) were the presence of each MSD (categorical: yes/no). Models with the highest number of confounding adjustments were extracted. Where analyses addressed the reverse association, the outcome was presence of PD or probable PD (any, Clusters, and/or specific PDs). The odds ratio (OR) and 95% confidence interval (95% CI) were the principal summary measures. For regression analyses, b values were used as the summary measure.

#### 2.4.3 Reporting of results

The MOOSE and PRISMA guidelines were considered for the reporting of results (40). The characteristics and results from individual studies, including critical appraisal scores, are presented in **Tables 2**, **3**, respectively. We determined levels of evidence for associations based on an adapted method previously published by Lievense et al. in the rheumatological setting (41) and in other published reviews (42, 43). This is presented in **Box 1** (below). There were too few studies with appropriately comparable study populations and groupings of PD to undertake meta-analyses.

**Table 3:** Summary of associations for included studies.

Citation and country	Study and population characteristics			Analytic approach	Summary of main findings
	Study design (data collection/follow-up period)	Study population; sample size (*n*)	Mean age (SD)/median(IQR)/age range Sex: % female		
Personality disorders and arthritis					
El-Gabalawy et al. (29) USA	Cross-sectional (NESARC Wave 2; 2004-2005)	Wave 2 NESARC participants N: 34,653	18+ Sex: 52.1%	Logistic regression, odds ratios (ORs) and 95% confidence intervals (CIs) • Adjusted for sex, ethnicity, education, marital status, income, age, lifetime mood disorder, lifetime anxiety disorder, lifetime substance disorder, and lifetime personality disorders (PDs) except borderline PD (i.e., antisocial, avoidant, dependent, obsessive–compulsive, paranoid, schizoid, histrionic, schizotypal, and narcissistic)	• Compared to those without, borderline PD was associated with increased odds of past-year arthritis (OR 1.56, 95% CI = 1.31, 1.85)
Goldstein et al. (30) USA	Cross-sectional (NESARC Wave 1; 2001–2002)	Wave 1 NESARC participants N: 43,093	48 (13.3) Sex: nr	Logistic regression, ORs, and 95% CI • Adjusted for age, sex, race/ethnicity, marital status, education, past-year personal income, health insurance coverage, region and urbanicity of respondent residence, body mass index, comorbid, lifetime diagnoses of nicotine dependence, any mood, anxiety, alcohol use, and drug use disorders, pathological gambling, lifetime PDs except antisocial PD (i.e., avoidant, dependent, obsessive–compulsive, paranoid, schizoid, and histrionic), average daily ounces of ethanol during period of heaviest lifetime drinking, frequency of use of most frequently used drug during period of heaviest lifetime use, and number of cigarettes smoked per day during most recent year of smoking	Compared to those with no history of antisocial behavioral syndromes: • Antisocial PD in men was associated with increased odds of past-year arthritis (OR 2.20, 95% CI = 1.69, 2.76) • Antisocial PD in women was associated with increased odds of past-year arthritis (OR 1.40, 95% CI = 1.03, 1.96) • Antisocial behavioral syndromes in men was associated with increased odds of past-year arthritis (OR 1.40, 95% CI = 1.20, 1.73) • Antisocial behavioral syndromes in women were not significantly associated with past-year arthritis (OR 1.20, 95% CI = 0.95, 1.42) • History of conduct disorder (only) in men was not significantly associated with past-year arthritis (OR 1.30, 95% CI = 0.66, 2.34) • History of conduct disorder (only) in women was not significantly associated with arthritis (OR 0.50, 95% CI = 0.26, 1.04)
McWilliams et al. (31) USA	Cross-sectional (NESARC Wave 1; 2001–2002)	Wave 1 NESARC participants N: 43,093	Aged 18+ Sex: nr	Logistic regression, ORs, and 95% CI • Adjusted for gender, marital status, income, age, and the presence of 1 or more health conditions other than arthritis, 1 or more past-year depressive disorders, 1 or more past-year anxiety disorders, and 1 or more past-year alcohol- or substance-related disorders	Compared to those without, past-year arthritis was associated with increased odds of: • Antisocial PD (OR 2.06, 95% CI = 1.72, 2.48) • Avoidant PD (OR 1.62, 95% CI = 1.27, 2.06) • Obsessive-compulsive PD (OR 1.41, 95% CI = 1.23, 1.62) • Paranoid PD (OR 1.40, 95% CI = 1.17, 1.67) • Schizoid PD (OR 1.79, 95% CI = 1.48, 2.17) • Histrionic PD (OR 1.80, 95% CI = 1.36, 2.39) Past-year arthritis was not significantly associated with dependent PD (1.49, 95% CI = 0.82, 2.70)
McWilliams & Higgins (28), USA	Cross-sectional (NCS Part II; 2001–2003)	Community-based respondents enrolled in Part II of the NCS-R N: 5,692	Aged 18+ Sex: nr	Regression, *b* values • Adjusted for sex, marital status, race, age, education level, past-year mood disorders, anxiety disorders, and externalizing disorders	• Compared to those without, lifetime arthritis was associated with higher borderline PD symptomology (0.19, p ≤ 0.01)
Powers & Oltmanns (32), USA	Cross-sectional (SPAN; dates nr)	Community-based residents aged 55–64 years enrolled in the SPAN N: 1,051	59.4 (2.7) Sex: 53%	Logistic regression, ORs, and 95% CI • Adjusted for: sex, age, race, marital status, education, any lifetime MDD, alcohol dependence or drug dependence, and any PD except borderline PD • BMI as “mediator”	• Compared to those without, interviewer-reported borderline PD features was associated with increased odds of arthritis (OR 2.64, 95% CI = 1.06–6.57) • Compared to those without, informant-reported borderline PD features was associated with increased odds of arthritis (OR 1.76, 95% CI = 1.05–2.95) • Compared to those without, self-reported borderline PD features were not significantly associated with increased odds of arthritis (1.41, 95% CI = 0.74, 2.68) • Full mediating effect of body mass index (BMI) on the association between borderline PD features and all three sources of PD assessment
Quirk et al. (38) USA	Cross-sectional (NESARC pooled; Wave 1 2001–2002 and Wave 2 2004–2005)	Wave I and 2 NESARC participants N: 34,653	Aged 20+ Sex: 52.1%	Logistic regression, ORs, and 95% CI • Adjusted for sex, race/ethnicity, marital status, education, income, age, past-year mood, anxiety and substance use disorders	Compared to those without, PD Clusters were associated with increased odds of past-year arthritis among people of all ages: • Cluster B PDs (OR 1.38, 95% CI = 1.18, 1.60) • Cluster C PDs (OR 1.35, 95% CI = 1.08, 1.69) Compared to those without, specific PDs were associated with increased odds of past-year arthritis among people of all ages: • Paranoid PD (OR 1.28, 95% CI = 1.02, 1.61) • Antisocial PD (OR 1.61 95% CI = 1.24, 2.07) • Borderline PD (OR 1.59, 95% CI = 1.27, 1.98) • Avoidant PD (OR 1.47, 95% CI = 1.11, 1.96) In subgroup analyses among those under <55 years, PDs were associated with increased odds of past-year arthritis for those with: • Any PD compared to without (OR 1.36, 95% CI = 1.13, 1.64) • Cluster A PDs compared to without (OR 1.39, 95% CI = 1.11, 1.75) • Schizoid PD compared to without (OR 1.62, 95% CI = 1.16, 2.26) • Schizotypal PD compared to without (OR 1.58, 95% CI = 1.18, 2.13) • Obsessive–compulsive PD compared to without (OR 1.41, 95% CI = 1.12, 1.79) In subgroup analyses among those ≥55 years, PDs were associated with increased odds of past-year arthritis for those with: • Any PD (OR 1.22, 95% CI = 1.03, 1.43) • Cluster C PDs (OR 1.28, 95% CI = 1.02, 1.61) PDs were not significantly associated with arthritis for all ages with: • Dependent PD (OR 1.39, 95% CI = 0.68, 2.85) • Histrionic PD (OR 1.17, 95% CI = 0.81, 1.7) • Narcissistic PD (OR 1.19, 95% CI = 0.98, 1.44) PD Clusters and specific PDs were not significantly associated with arthritis for those over 55 years with: • Any Cluster A PD (OR 1.15, 95% CI = 0.89, 1.48) • Schizoid PD (OR 1.18, 95% CI = 0.83, 1.68) • Schizotypal PD (OR 1.16, 95% CI = 0.78, 1.72) • Obsessive-compulsive PD (OR 1.11, 95% CI = 0.85, 1.45) Any C Cluster PD was not significantly associated with arthritis among those under 55 years (OR 1.20, 95% CI = 0.93, 1.54).
Quirk et al. (33) Australia	Cross-sectional (GOS; 2011–2014)	Community-based women enrolled in the GOS in southeastern Australia N: 765	56.8 (42.7–68.9) Sex: 100%	Logistic regression, ORs, and 95% CI • Adjusted for age, BMI, SES, physical activity, smoking status, and psychiatric disorder (ever), and interactions of IRSAD* smoking status, physical activity* psychiatric disorder (ever)	• Cluster B PDs were associated with increased odds of lifetime arthritis (OR 4.25, 95% CI = 1.34, 13.44) Grouped PDs were not significantly associated with arthritis: • Any PD (OR 1.07, 95% CI = 0.65, 1.75) • Cluster A PDs (OR 1.36, 95% CI = 0.53, 3.45) • Cluster C PDs (OR 1.04, 95% CI = 0.62, 1.75)
Personality disorders and spinal pain					
McWilliams & Higgins (28), USA	Cross-sectional (NCS Part II; 2001–2003)	Community-based respondents enrolled in Part II of the NCS-R N: 5,692	Aged 18+ Sex: nr	Regression, b values • Adjusted for sex, marital status, race, age, education level, past-year mood disorders, anxiety disorders, and externalizing disorders	• Compared to those without, past-year spinal pain was associated with higher borderline PD symptomology (0.38, p ≤ 0.001) • Compared to those without, remitted spinal pain was associated with higher borderline PD symptomology (0.31, p ≤ 0.001) • No significant differences in borderline PD symptomatology between past-year spinal pain and remitted spinal pain
Personality disorders and fibromyalgia/muscular pain					
Olssøn & Dahl (37), Norway	Case-control (HUBRO; May 2000–September 2001)	Community-based respondents to the HUBRO study health Survey N: 2,214 Cases: 369 Controls: 1,845	Aged 30+ Sex: 48%	χ^2^ -tests; 2 × 2 contingency tables were calculated as effect sizes with Cohen’s *d* • Matched on age and gender • Adjusted for education and employment status	*Personality problems predicting fibromyalgia/muscular pain:* • 33% of people with personality problems had past-year muscular pain versus 22% of controls (p < 0.001) • 4% of people with personality problems had lifetime fibromyalgia versus 2% of controls (p < 0.001)
				Logistic regression, ORs and 95% CI • Matched on age and gender • Adjusted for education and employment status	*Fibromyalgia/muscular pain predicting personality problems:* • Past-year *muscular* pain was associated with increased odds of personality problems (OR 1.4, 95% CI = 1.1, 2.9) • Lifetime fibromyalgia was not significantly associated with personality problems
Olssøn & Dahl (34), Norway	Case-control (HUBRO; May 2000–September 2001)	Community-based respondents to the HUBRO study health Survey N: 280 (cases) N: 1,400 (controls)	Aged 30+ Sex: 65%	χ^2^ tests; 2 × 2 contingency tables were calculated as effect sizes with Cohen’s *d* • Matched on age and gender Adjusted for education and employment status	*Personality problems predicting muscular pain:* • 37% of people with probable avoidant PD had muscular pain versus 20% of controls (p < 0.001; ES 3.8)
				Logistic regression, ORs and 95% CI • Matched on age and gender Adjusted for demographic impairment, somatic impairment, frequent use of analgesics, ≥4 past-year visits to GP, mental impairment, insomnia affecting work ability, frequent use of psychotropics, ≥4 past-year visits to psychiatrist/psychologist	*Muscular pain predicting personality problems:* • Muscular pain was not significantly associated with probable avoidant PD
Sleurs et al. (39) USA	Cross-sectional (NESARC Wave 3 2012–2013)	N: 36,309	18+ Sex: 51.16% (no fibromyalgia); 87.48% (fibromyalgia present)	Logistic regression, ORs, and 95% CI • Adjusted for sex, race, nativity, age, education, income, marital status, urbanicity and region	Compared to those without, fibromyalgia was associated with increased odds of PDs: • Any PD (OR 2.91, 95% CI = 2.32, 3.65) • Schizotypal PD (OR 3.57, 95% CI = 2.72, 4.67) • Borderline PD (OR 2.86, 95% CI = 2.32, 3.52) • Antisocial PD (OR 3.06, 95% CI = 1.93, 4.85)
Personality disorders and bone health					
Williams et al. (25) Australia	Cross-sectional (GOS; 2011–2014)	Community-based women enrolled in the GOS in southeastern Australia N: 696	56.8 (42.7–68.9) Sex: 100%	χ^2^ tests	• 6.1% of people with any PD had osteoporosis versus 8.7% without (p = 0.335)
				Regression, b values • Adjusted for age and sex (female only)	• Cluster A PDs were associated with lower BMD at the hip (−0.057, p = 0.027) • Trend for association between Cluster A PDs and lower total body BMD (−0.037, p = 0.056) No significant association between: • Clusters B and total body BMD (p > 0.05) • Cluster B PDs and hip BMD (p > 0.05) • Cluster C PDs and total body BMD (p > 0.05) • Cluster C PDs and hip BMD (p > 0.05) • C PDs not significantly associated with total body or hip BMD (p > 0.05) • Cluster A PDs and spine BMD (p > 0.05) • Cluster B PDs and spine BMD (p > 0.05) • Cluster C PDs and spine BMD (p > 0.05)

Box 1 **Criteria for levels of evidence derived from Lievense et al. (41).**

Strong evidence

Generally consistent findings in:

Multiple high-quality cohort studies

Moderate evidence

Generally consistent findings in:

1 high-quality cohort study and ≥2 high quality case-control studies

≥3 high-quality case-control studies

Limited evidence

Generally consistent findings in:

1 high-quality cohort study

<2 high-quality cross-sectional, case-control studies

Inconsistent evidence

If ≤75% studies reported consistent findings

No evidence

No evidence was identified

## 3 Results

The results of the study identification and selection process are presented in **Figure 1**. Via the EBSCOhost platform, the searches from APA PsycINFO yielded 143 citations (additional n = 1 from the updated search), 94 from CINAHL Complete (additional n = 5 from updated search), 948 from Embase (additional n = 114 from updated search), and 236 from Medline Complete (additional n = 16 from updated search). After duplicates (n = 297 total) were removed, the total search yield included 1,260 citations. Of the 1,260 citations screened, 23 underwent full-text review, and 12 were excluded with reasons. No further citations were yielded from searching the other evidence sources. Thus, 11 peer-reviewed published articles were included in this review.

### 3.1 Characteristics

The characteristics of the included studies and summary of results are presented in **Tables 2**, **3**. The reports of the identified studies were published between 2008 and 2020. Of the 11 studies included, nine were cross-sectional and two were case control. Seven out of the 11 studies were conducted in the United States (US) (28–32, 38, 39), with two studies each from Norway (34, 37) and Australia (25, 33). Epidemiological data sources included Waves I/II/III of the National Epidemiologic Survey on Alcohol and Related Conditions (NESARC) (29–31, 38, 39), the National Comorbidity Survey—Part II (28), and The St. Louis Personality and Aging Network (SPAN) study (32) each from the US; the Oslo Health Study (HUBRO) from Norway (34, 37), and the Geelong Osteoporosis Study (GOS) located in Australia (25, 33). Participant samples ranged from n = 696 in the GOS study from Australia (25) to n = 43,093 in two studies utilizing data from the NESARC (30, 31); two studies utilized samples comprising only women (25, 33).

Eight studies used semi-structured interviews to identify PDs including the Alcohol Use Disorder and Associated Disabilities Interview Schedule-IV (AUDADIS-IV)/AUDADIS-5 (29–31, 38, 39) performed by trained lay interviewers, the Structured Interview for DSM-IV Personality (SIDP-IV) (32) and the Structured Clinical Interview for DSM-IV (SCID-II) by trained interviewers (25, 33). Two studies identified probable PDs using The Iowa Personality Disorder Screen (IPDS) (34, 37) with another using the borderline PD scale from the International Personality Disorder Examination (IPDE) Screening Questionnaire (28). Majority (10/11) of studies utilized self-report of MSDs including arthritis (28, 29, 31–33, 38), fibromyalgia/muscular pain (37, 39), and spinal pain (28). One study determined osteoporosis (yes/no) by a BMD T-score of <−2.5 at the spine and/or hip using dual-energy X-ray absorptiometry (Prodigy; GE Lunar, Madison, WI, USA) (25). There were no studies identified that classified PD using measures based on the ICD-11 classification system.

The median critical appraisals score was 6, and thus, the reporting quality of most studies were considered high. In terms of the cross-sectional studies, the most common reason for not achieving a total sum score of 7 concerned the quality of describing the study population and setting, and/or the use of a reliable and valid measure of the study exposure (i.e., use of an adapted measure to assess PD). Regarding the case-control studies (whereby a total possible score is 9), a lack of information or unclear descriptions of the methodology (i.e., appropriate matching of cases and controls, validity and reliability of the exposure measure, and details concerning the appropriateness of the statistical analyses) resulted in a lower critical appraisal score.

### 3.2 Descriptive synthesis

The following sections present the findings from the descriptive synthesis and evidence gap analysis in text and tables. Specifically, **Table 3** presents the results from all individuals studies/analyses with the evidence gap analysis shown in **Supplementary Tables 7**-**14**.

#### 3.2.1 Personality disorders and arthritis

In the literature, associations between PDs and arthritis were examined in seven cross-sectional studies (28–33, 38). Of these, four utilized data from the NESARC (29–31, 38); the remaining three presented analyses from Part II of the NCS (28), the SPAN (32), and the GOS (33).

In Wave I of the NESARC, Goldstein et al. examined antisocial PD, antisocial PD features, and history of conduct disorder and the association with past-year arthritis, compared to people with no history of these PDs/features, and according to sex (30). Men and women with antisocial PD had increased odds of arthritis compared to those without a history (30). Separately, in Wave II of the NESARC, El-Gabalawy et al. reported an association between borderline PD and increased odds of past-year arthritis (29). In a further study using data from both waves I and II, Quirk et al., examined all PDs and PD Clusters in relation to past-year arthritis considering the role of age in these associations (38). Across all specific PDs and PD groupings, individuals with grouped Cluster B PDs had the highest odds of arthritis compared to those without these PDs. In addition, for those in the younger age group (*under 55 years*), any PD, schizoid, schizotypal, and obsessive–compulsive PDs were each associated with increased odds of arthritis compared to younger individuals without these PDs (38). Of the analyses deriving from the NESARC, all accounted for pertinent sociodemographics and at least mood, anxiety, and substance use disorders (29, 30, 38). In addition, the separate analyses presented in the reports by El-Gabalawy et al. and Goldstein et al. also adjusted for PDs other than the exposure (29, 30). Finally, in the GOS, Quirk et al. reported increased odds of a history of arthritis among women with Cluster B PDs compared to women without these PDs after adjustment for sociodemographic information, lifestyle behaviors, lifetime history of any mood, anxiety, substance use, or eating disorders (and the interactions of these) (33).

In terms of the reverse association, in Wave I of the NESARC, McWilliams et al. examined associations between past-year arthritis and odds of several specific PDs (avoidant, dependent, obsessive–compulsive, paranoid, schizoid, histrionic, and antisocial PDs) (31). Compared to people without, people with arthritis had increased odds of paranoid, schizoid, histrionic, antisocial, avoidant, and obsessive–compulsive PDs—analyses accounted for pertinent sociodemographic factors (31). In addition, in Part-II of the NCS, McWilliams reported that, compared to people without, those with lifetime arthritis had higher BPD symptomatology scores after accounting for sociodemographics, past-year mood disorders, anxiety disorders, and externalizing disorders (28). Separately, in the SPAN, Powers et al., reported increased odds of arthritis among people with interviewer- and informant-rated borderline PD features, but not self-reported borderline PD features, among adults aged 55 to 64 years. However, the association between all three types of assessment modes (of borderline PD features) and arthritis was fully mediated by BMI (32).

Also, non-significant findings for associations between PDs and arthritis were also observed. Analyses deriving from Wave I of the NESARC revealed that dependent PD was not significantly associated with greater odds of arthritis (31) with further analyses from pooled Waves I and II of the NESARC showing non-significant results for associations between histrionic and narcissistic PDs and arthritis (38). In subgroup analyses deriving from Wave I of the NESARC, antisocial behavioral syndromes were not significantly associated with arthritis among women, nor was a history of conduct disorder for both women and men (30). Also in the NESARC (Waves I and II pooled), those aged over 55 years, having any grouped Cluster A PDs, or the specific PDs of schizoid, schizotypal, or obsessive–compulsive were not significantly associated with greater odds of arthritis; being in the younger age group (under 55 years) and having any C Cluster PD was not significantly associated with arthritis (38). Finally, in the GOS (women only), any PD grouped, Cluster A PDs, and Cluster C PDs were not significantly associated with arthritis (25).

#### 3.2.2 Personality disorders and spinal pain

In analyses utilizing data from Part-II of the NCS, McWilliams et al. examined associations between a history of spinal pain, as the exposure, and borderline PD symptomatology (28). Compared to people without, people with past-year spinal pain had higher borderline PD symptomatology following adjustment for pertinent sociodemographics, past-year mood disorders, anxiety disorders, and externalizing disorders (28). This association was similarly observed among people with remitted spinal pain compared to people without remitted spinal pain (28). However, in further analyses, people with past-year spinal pain did not report significantly different borderline PD symptomatology compared to people who had remitted (28).

#### 3.2.3 Personality disorders and fibromyalgia/muscular pain

Two separate reports of studies utilizing data from the HUBRO (34, 37) and one from Wave III of the NESARC examined associations between PDs and fibromyalgia/muscular pain (39). Olssøn and Dahl examined both directions of associations between personality problems and fibromyalgia and muscular pain, respectively. First, a higher proportion of people with probable PD had past-year muscular pain and lifetime fibromyalgia compared to controls (age–gender matched), respectively (37). Moreover, muscular pain, but not fibromyalgia, was associated with increased odds of probable PD after additional adjustments for education and employment status (37). Next, Olssøn and Dahl examined avoidant personality problems specifically and found that a higher proportion of people with probable avoidant PD had muscular pain compared to controls (age and gender matched). In reverse, muscular pain was not significantly associated with probable avoidant PD (adjusted further for education and employment status) (34). Elsewhere, in Wave 3 of the NESARC, Sleurs et al. reported that compared to those without, people with fibromyalgia have increased odds of “any” PD as well as each schizotypal, borderline, and antisocial PDs (39); analyses accounted for sociodemographic information.

#### 3.2.4 Personality disorders and bone health

In one study using the GOS data, associations between any PD and osteoporosis among women were not statistically significant (25). However, in analyses examining associations between PD Clusters and BMD, women with Cluster A PDs, but not Cluster B or C PDs, had lower hip BMD (i.e., poorer bone health) compared to women without these PDs (25). Women with Cluster A, B, or C PDs did not have significantly reduced total body BMD or spine BMD.

#### 3.2.5 Levels of evidence

Overall, our evidence gap analysis (see **Supplementary Tables 7**-**14**) revealed that there was either conflicting or limited evidence for associations between PDs and arthritis, spinal pain, fibromyalgia, and BMD. In addition, there was currently no evidence (i.e., these studies have not yet been conducted) for associations between a range of specific PDs in regard to these MSDs especially concerning BMD.

## 4 Discussion

This review had two aims: 1) to evaluate the evidence for associations between PDs and MSDs including arthritis, back/spinal pain, fibromyalgia/muscular pain, and aspects of bone health; to explore potential sources of heterogeneity in the results including study and population characteristics and the assessment of PD and 2) ascertain the quality and levels of evidence for these associations.

Overall, our descriptive synthesis revealed that there is currently limited and conflicting levels of evidence for associations between PDs and each of the MSDs. While 10 of the 11 studies reviewed were considered of “high quality,” we identified several methodological factors that might contribute to the limited evidence base for these associations. First, the evidence identified was derived primarily from cross-sectional studies, and thus, evidence from case-control and cohort studies are needed to ascertain moderate or strong evidence for these associations if evident. In addition, we identified heterogenous groupings of PD as the exposure of interest—particularly for “any” PD—and the extent to which the full range of specific PDs were examined also varied. Considering the relatively small number of studies identified, the numerous PD groupings, and number of participants, the comparisons of alike studies were limited.

In terms of specific MSD outcomes, we identified conflicting evidence for associations between Cluster A PDs and arthritis. To illustrate, in the NESARC, paranoid PD among all ages was associated with increased odds of arthritis with schizoid and schizotypal PDs each being associated with arthritis among those *under* 55 years (not among those *over 55 years.*) When examined as a “Cluster” in relation to arthritis, the association with these PDs were attenuated (although remained significant). Separately, in the GOS, these PDs were examined as a Cluster among women with an average age of approximately 57 years; in this study, Cluster A PDs were not significantly associated with increased odds of arthritis. Similarly, in pooled Waves 1 and 2 of the NESARC, Quirk et al. reported no significant associations between Cluster C PDs (grouped) and increased odds of arthritis among individuals of all ages or those *aged under 55 years*. However, in separate analyses from the same report, people *over the age of 55 years* with Cluster C PDs were found to have significantly increased odds of arthritis; in the GOS, Cluster C PDs were not significantly associated with arthritis. Thus, the conflicting evidence for associations between Cluster A and C PDs and arthritis appears to be influenced by how these PDs are grouped and the nature of the study populations including age and sex factors.

There is a need to improve the evidence base on population-based associations between PD and MSDs. This expansion would contribute to a more comprehensive understanding of the interplay between these conditions and encourage investigation into potential shared etiological pathways, common risk factors, and consideration of therapeutic implications.

### 4.1 Mechanisms

In a recent scoping review, we highlighted that the mechanisms underlying potential associations between PDs and MSDs could be understood from the perspective of the biopsychosocial model (9). To date, biopsychosocial models have been employed to understand how the interaction of psychological, social, and biological factors operate in the etiology and maintenance of pain specifically (44–46). Thus, the biopsychosocial models provides a transdiagnostic lens to conceptualize how mechanisms might operate in concert across PD and MSDs.

To illustrate, exposure to early stress, trauma, and other forms of adversities in childhood and adolescence—critical periods for personality development and the acquisition of adaptive self-regulatory processes—are understood to alter stress mechanisms. These mechanisms are understood to have a role in vulnerability to PD via alterations in the hypothalamic–pituitary–adrenal (HPA) axis and morphological changes in brain areas involved in the stress response (47). Childhood adversity and types of trauma have been separately linked to PD (48–50) and to MSDs including arthritis (51) and chronic back pain (52). Alterations in the stress response can also lead to increased vulnerability of a range of physical disorders (45, 46). Moreover, a recent study reported associations between childhood adversity/trauma and borderline PD symptomatology among patients with chronic pain including pain related to MSDs (53).

Of interest, we observed several analyses showing associations between Cluster B PDs and arthritis specifically. There is evidence that stress is associated with emotional dysregulation, which often presents as hypersensitivity and reactivity and difficulty coping among people with these PDs (54). Stress is also associated with immune dysregulation and inflammation in the body (55). Acute stress arising as a symptom or consequence of PD may “trigger” or exacerbate MSDs that have inflammatory origins such as conditions of the joints through immune dysregulation and inflammatory responses. Separately, Turk and Monarch explained that intense emotions, such as anger, may interfere with help seeking and willingness to engage with treatment recommendations among people with psychiatric disorders and thus minimize the opportunity for effective rehabilitation (44). Others have highlighted the complexity of the experience of pain among people with borderline PD. For example, it is suggested that, as a consequence of self-regulation difficulties, the experience of endogenous pain may be felt as more intense and less tolerable, compared to self-inflicted pain; pain is also understood to serve an affect-regulating function for people with borderline PD, which can, in turn, lead to pain attenuation and tolerance (56, 57). To substantiate, previous experimental research has uncovered that modulating pain can regulate affect in patients with PD (58), which is reduced following treatment with DBT (59).

In this review, there was mixed evidence for associations between “Cluster C” PDs and MSDs. “Cluster C” PDs are traditionally conceptualized to manifest the behaviors of extreme avoidance of social interaction, which are motivated by feelings and fears of perceived inadequacy and rejection (5). People with features of these disorders may experience anxiety associated with pain leading to types of avoidance coping or safety behaviors. These may manifest externally through the avoidance of social interactions/situations or internally to avoid experiencing distressing thoughts, feelings, or sensations concerning pain (60). In addition, a recent review has summarized that features of these disorders tend to be more stable over time (61), and thus, avoidance styles of coping may became more entrenched and operate in the maintenance of comorbidities.

Given the plausible biopsychosocial factors linking PDs and MSDs, we have previously suggested that a multidisciplinary approach to identifying and treating comorbidities in clinicals settings may be needed (9). However, there is a lack of research into, and targeted measures and interventions for, each psychiatric and musculoskeletal medicine settings to identify and concurrently manage PDs and MSDs.

### 4.2 Strengths and limitations of this review

In terms of strengths, we developed and implemented a comprehensive search strategy including a thorough search for evidence from gray literature sources, which enabled a complete synthesis of the existing literature. We reported on the current levels of evidence for associations between PDs and revealed where evidence gaps exist, which will prompt and guide further research on this topic. We also provided a conceptual description of the possible mechanisms that might underly associations between PDs and MSDs—ideas to be further investigated and empirically tested in clinical settings.

In terms of the limitations, most notably was the paucity of data for each outcome. The evidence gap analysis revealed “no evidence” for a number of specific PDs and MSDs particularly in relation to BMD. Thus, the authors suggest that a call to action is needed to address the evidence gap and understand if people with PDs may be more susceptible to MSDs for which clinical monitoring and management may be required. In addition, the outcomes were chiefly assessed using self-reported measures (except for one study, which also utilized clinical measures of BMD). Recent evidence from a population-based study comparing self-report to register data in Finland (women only) reported that self-reported measures may be insufficient for accurately identifying MSDs (62). Thus, MSDs may be underrepresented in the reviewed studies.

A significant proportion of the studies utilized data from Waves of the NESARC (5 of the 11 studies), and there were few opportunities to synthesize the evidence examining associations between PDs and MSDs from varied studies and settings. In addition, this review included eligible analyses, which involved multiple comparisons of the same data source, which may be considered a limitation. Similarly, in terms of the assessment of PD, we note that multiple comparisons, that is, the number of possible PD groupings (i.e., 3 PD Clusters, 10 specific PDs, and pooled PDs) in relation to MSDs within and across studies, has limited what conclusions can be drawn about associations between PDs and MSDs. It is suggested that derivation of an “any PD” may assist in overcoming this methodological issue in future research. However, it is acknowledged that traditionally, these specific PDs vary in clinical presentation and severity, and thus, meaningful, and sensitive data may be lost if any one or more PDs are grouped as a unitary variable.

In terms of study designs, the available evidence was derived chiefly from cross-sectional studies, and no longitudinal studies were identified. Therefore, it is not presently possible to determine the direction of causality among associations between PDs and MSDs. There was also insufficient evidence sources to complete planned meta-analyses on associations between PDs and MSDs.

Therefore, considerably expanding the evidence base on the longitudinal course of PD and MSDs would facilitate a thorough exploration of the underlying mechanisms and understanding of potential clinical implications. For example, a burgeoning evidence base might lead to a greater focus in both psychiatric and musculoskeletal medicine settings to identify and concurrently manage PDs and MSDs. In addition, an evolving evidence base would allow for a deeper and more nuanced interpretation of synthesized evidence in future reviews on this important topic. Future research should also consider the application of the ICD-11 classification of PD in relation to these comorbidities.

## 5 Conclusions

To conclude, the quality of most studies included in this review that examined associations between PD and MSDs in general population adults was high. However, the results demonstrated limited and conflicting evidence for these associations—in part due to a lack of comparable cross-sectional studies and no detected longitudinal evidence, which is now needed.

## Data availability statement

The original contributions presented in the study are included in the article/**Supplementary Material**. Further inquiries can be directed to the corresponding author.

## Author contributions

SQ: Conceptualization, Methodology, Writing – original draft, Writing – review & editing. HK: Conceptualization, Methodology, Writing – original draft, Writing – review & editing. RH: Writing – original draft, Writing – review & editing. MM: Methodology, Writing – original draft, Writing – review & editing. AS: Methodology, Writing – original draft, Writing – review & editing. JH: Writing – original draft, Writing – review & editing. LW: Conceptualization, Methodology, Supervision, Writing – original draft, Writing – review & editing.
