# Supplementary material for: A systematic review of personality and musculoskeletal disorders: evidence from general population studies
Source: Front Psychiatry. 2024 May 21;15:1288874. doi: 10.3389/fpsyt.2024.1288874 (PMC11148376; doi:10.3389/fpsyt.2024.1288874)
Supplement: Supplementary file 1 [file DataSheet_1.docx]

Supplementary Material

A systematic review of personality and musculoskeletal disorders: evidence from general population studies

Shae E Quirk^1-3*^, Heli Koivumaa-Honkanen^2,3^, Risto J Honkanen^2,3^, Mohammadreza Mohebbi^4^, Amanda L Stuart^1^, Jeremi Heikkinen^2,3^, Lana J Williams^1,6^

**Supplementary Table 1:** Search for Medline Complete

| Search # | Search terms |
| --- | --- |
| S10 | S9 AND 19038772 OR 23328328 OR 20508177 OR 18396181 OR 17890160 OR 22686464 OR 25314916 OR 26928343 OR 22146705 OR 33363487) |
| S9 | S7 AND S8 |
| S8 | S3 OR S4 OR S5 OR S6 |
| S7 | S1 OR S2 |
| S6 | MH ("Fibromyalgia" OR “Myalgia” OR “Musculoskeletal Pain”) OR TI (fibromyal* OR fibromyalgia OR "musc* pain" OR “muscle aches” OR “muscle soreness”) OR AB (fibromyal* OR fibromyalgia OR "musc* pain" OR “muscle aches” OR “muscle soreness”) |
| S5 | MH ("Osteoporosis+") OR TI (osteoporosis OR osteopenia OR bone) OR AB (osteoporosis OR osteopenia OR bone) |
| S4 | MH ("Back Pain+" OR “Neck Pain") OR TI (“back pain” OR backache* OR “low back pain” OR “neck pain” OR "lumbar pain" OR "spinal pain" OR “spine pain” OR "spinal stenosis" OR "lumbar stenosis" OR "intervertebral disc displacement" OR “disc herniation” OR sciatica) OR AB (“back pain” OR backache* OR “low back pain” OR “neck pain” OR "lumbar pain" OR "spinal pain" OR “spine pain” OR "spinal stenosis" OR "lumbar stenosis" OR "intervertebral disc displacement" OR “disc herniation” OR sciatica) |
| S3 | MH (Arthritis+) OR TI (arthritis OR osteoarthritis OR "osteo-arthritis" OR "osteoarthritic") OR AB (arthritis OR osteoarthritis OR "osteo-arthritis" OR "osteoarthritic") |
| S2 | MH ("Personality Disorders+") |
| S1 | TI ((personality OR borderline) N2 (disorder* OR dysfunction* OR pathology OR feature* OR symptom*)) OR AB ((personality OR borderline) N2 (disorder* OR dysfunction* OR pathology OR feature* OR symptom*)) |

Note: Search modes = Boolean/Phrase. Search fields = search in abstract field (AB); search in MeSH/Index Term field (MH); search title field (TI); explode (+)

**Supplementary Table 2**: Search for CINAHL Complete

| Search # | Search terms |
| --- | --- |
| S9 | S7 AND S8 |
| S8 | S3 OR S4 OR S5 OR S6 |
| S7 | S1 OR S2 |
| S6 | MH ("Fibromyalgia" OR “Muscle Pain”) OR TI (fibromyal* OR fibromyalgia OR "musc* pain" OR “muscle aches” OR “muscle soreness”) OR AB (fibromyal* OR fibromyalgia OR "musc* pain" OR “muscle aches” OR “muscle soreness”) |
| S5 | MH ("Osteoporosis+") OR TI (osteoporosis OR osteopenia OR bone) OR AB (osteoporosis OR osteopenia OR bone) |
| S4 | MH ("Back Pain+" OR “Neck Pain") OR TI (“back pain” OR backache* OR “low back pain” OR “neck pain” OR "lumbar pain" OR "spinal pain" OR “spine pain” OR "spinal stenosis" OR "lumbar stenosis" OR "intervertebral disc displacement" OR “disc herniation” OR sciatica) OR AB (“back pain” OR backache* OR “low back pain” OR “neck pain” OR "lumbar pain" OR "spinal pain" OR “spine pain” OR "spinal stenosis" OR "lumbar stenosis" OR "intervertebral disc displacement" OR “disc herniation” OR sciatica) |
| S3 | MH (Arthritis+) OR TI (arthritis OR osteoarthritis OR "osteo-arthritis" OR "osteoarthritic") OR AB (arthritis OR osteoarthritis OR "osteo-arthritis" OR "osteoarthritic") |
| S2 | MH ("Personality Disorders+") |
| S1 | TI ((personality OR borderline) N2 (disorder* OR dysfunction* OR pathology OR feature* OR symptom*)) OR AB ((personality OR borderline) N2 (disorder* OR dysfunction* OR pathology OR feature* OR symptom*)) |

Note: Search modes = Boolean/Phrase. Search fields = search in abstract field (AB); search in MeSH/Index Term field (MH); search title field (TI); explode (+)

**Supplementary Table 3:** Search for APA PsycInfo

| Search # | Search terms |
| --- | --- |
| S9 | S7 AND S8 |
| S8 | S3 OR S4 OR S5 OR S6 OR S7 OR S8 |
| S7 | S1 OR S2 |
| S6 | DE ("Fibromyalgia") OR TI (fibromyal* OR fibromyalgia OR "musc* pain" OR “muscle aches” OR “muscle soreness”) OR AB (fibromyal* OR fibromyalgia OR "musc* pain" OR “muscle aches” OR “muscle soreness”) |
| S5 | DE ("Osteoporosis") OR TI (osteoporosis OR osteopenia OR bone) OR AB (osteoporosis OR osteopenia OR bone) |
| S4 | DE ("Back Pain") OR TI (“back pain” OR backache* OR “low back pain” OR “neck pain” OR "lumbar pain" OR "spinal pain" OR “spine pain” OR "spinal stenosis" OR "lumbar stenosis" OR "intervertebral disc displacement" OR “disc herniation” OR sciatica) OR AB (“back pain” OR backache* OR “low back pain” OR “neck pain” OR "lumbar pain" OR "spinal pain" OR “spine pain” OR "spinal stenosis" OR "lumbar stenosis" OR "intervertebral disc displacement" OR “disc herniation” OR sciatica) |
| S3 | DE ("Arthritis" OR "Rheumatoid Arthritis") OR TI (arthritis OR osteoarthritis OR "osteo-arthritis" OR "osteoarthritic") OR AB (arthritis OR osteoarthritis OR "osteo-arthritis" OR "osteoarthritic") |
| S2 | DE ("Personality Disorders" OR "Antisocial Personality Disorder" OR "Avoidant Personality Disorder" OR "Borderline Personality Disorder" OR "Dependent Personality Disorder" OR "Histrionic Personality Disorder" OR "Narcissistic Personality Disorder" OR "Obsessive Compulsive Personality Disorder" OR "Paranoid Personality Disorder" OR "Passive Aggressive Personality Disorder" OR "Sadomasochistic Personality" OR "Schizoid Personality Disorder" OR "Schizotypal Personality Disorder") |
| S1 | TI ((personality OR borderline) N2 (disorder* OR dysfunction* OR pathology OR feature* OR symptom*)) OR AB ((personality OR borderline) N2 (disorder* OR dysfunction* OR pathology OR feature* OR symptom*)) |

Note: Search modes = Boolean/Phrase. Search fields = search in abstract field (AB); search in Index Term field (DE); search title field (TI); explode (+)

**Supplementary Table 4:** Search for Embase

| Search # | Search terms |
| --- | --- |
| S15 | #3 AND #13 AND [embase]/lim AND [1990-2021]/py |
| S14 | #3 AND #13 |
| S13 | #4 OR #5 OR #6 OR #7 OR #8 OR #9 OR #10 OR #11 OR #12 |
| S12 | 'musculoskeletal pain'/exp |
| S11 | 'myalgia'/exp OR 'fibromyalgia'/exp |
| S10 | fibromyal*:ti,ab OR fibromyalgia:ti,ab OR 'musc* pain':ti,ab OR 'muscle aches':ti,ab OR 'muscle soreness':ti,ab |
| S9 | 'osteoporosis'/exp |
| S8 | osteoporosis:ti,ab OR osteopenia:ti,ab OR bone:ti,ab |
| S7 | 'backache'/exp OR 'low back pain'/exp OR 'neck pain'/exp |
| S6 | 'back pain':ti,ab AND otbackache*:ti,ab OR 'low back pain':ti,ab OR 'neck pain':ti,ab OR 'lumbar pain':ti,ab OR 'spinal pain':ti,ab OR 'spinal stenosis':ti,ab OR 'lumbar stenosis':ti,ab OR 'intervertebral disc displacement':ti,ab OR 'disc herniation':ti,ab OR sciatica:ti,ab |
| S5 | 'arthritis'/exp |
| S4 | arthritis:ti,ab OR osteoarthritis:ti,ab OR 'osteo-arthritis':ti,ab OR osteoarthritic:ti,ab |
| S3 | #1 OR #2 |
| S2 | 'personality disorder'/exp |
| S1 | ((personality OR borderline) NEAR/2 (disorder* OR dysfunction* OR pathology OR feature* OR symptom*)):ti,ab |

Note: Combine using And/Or. fields = search in Emtree (explode), abstract field (ab), and title (ti)

**Supplementary Table 5:** Search for CORDIS (grey literature)

| Search terms | Filtered by: | Results |
| --- | --- | --- |
| (/result/relations/categories/collection/code='deliverable','publication','exploitable' OR /result/relations/categories/collection/code='pubsum') AND ('personality disorder' AND 'arthritis' OR 'fibromyalgia' OR 'myalgia' OR 'osteoporosis' OR 'musculoskeletal pain' OR 'back pain' OR 'neck pain' OR 'spinal pain') | - Project deliverables - Project publications - Exploitable results - Report summaries | It yielded 686 |

**Supplementary Table 6:** Search for PROQUEST (grey literature)

| S6 | S1 AND (S2 OR S3 OR S4 OR S5) |
| --- | --- |
| S5 | Title(fibromyal* OR "musc* pain" OR “muscle aches” OR “muscle soreness”) OR Abstract(fibromyal* OR "musc* pain" OR “muscle aches” OR “muscle soreness”) |
| S4 | Title(osteoporosis OR osteopenia OR bone) OR abstract(osteoporosis OR osteopenia OR bone) |
| S3 | title(“back pain” OR backache* OR “neck pain” OR "lumbar pain" OR "spinal pain" OR “spine pain” OR "spinal stenosis" OR "lumbar stenosis" OR "intervertebral disc displacement" OR “disc herniation” OR sciatica) OR abstract(“back pain” OR backache* OR “neck pain” OR "lumbar pain" OR "spinal pain" OR “spine pain” OR "spinal stenosis" OR "lumbar stenosis" OR "intervertebral disc displacement" OR “disc herniation” OR sciatica) |
| S2 | Title(arthriti* OR "osteo arthriti*" OR osteoarthriti*) OR Abstract(arthriti* OR "osteo arthriti*" OR osteoarthriti*) |
| S1 | Title("personality disorder*") OR Abstract("personality disorder*") |

**Supplementary Table 7:** Evidence gap analysis of associations between PDs (exposure) and arthritis (outcome)

| **Type of PD/grouping** | **No evidence** | **Inconsistent Evidence** | **Limited Evidence** | | | | **Moderate Evidence** | **Strong Evidence** |
| --- | --- | --- | --- | --- | --- | --- | --- | --- |
|  |  | *If ≤75% studies reported consistent findings* |  | *Generally consistent findings in:* | | |  |  |
|  |  |  | 1 high-quality cross-sectional study | 2 high-quality cross-sectional studies | ≥ 3 high-quality cross-sectional studies | 1 or 2 high-quality case-control studies |  |  |
| Any PD |  |  |  |  |  |  |  |  |
| Probable PD |  |  |  |  |  |  |  |  |
| Cluster A PDs |  |  |  |  |  |  |  |  |
| Paranoid PD |  |  |  |  |  |  |  |  |
| *All* |  |  |  |  |  |  |  |  |
| *<55 years* |  |  |  |  |  |  |  |  |
| Schizoid PD |  |  |  |  |  |  |  |  |
| *<55 years* |  |  |  |  |  |  |  |  |
| Schizotypal PD |  |  |  |  |  |  |  |  |
| *<55 years* |  |  |  |  |  |  |  |  |
| Cluster B PDs |  |  |  |  |  |  |  |  |
| Antisocial PD |  |  |  |  |  |  |  |  |
| *All* |  |  |  |  |  |  |  |  |
| *Women* |  |  |  |  |  |  |  |  |
| *Men* |  |  |  |  |  |  |  |  |
| Antisocial behavioural syndromes |  |  |  |  |  |  |  |  |
| *Men* |  |  |  |  |  |  |  |  |
| *Women* |  |  | - ^*^ |  |  |  |  |  |
| Borderline PD |  |  |  | - ^†^ |  |  |  |  |
| Borderline PD features/symptoms |  |  | - * |  |  |  |  |  |
| Histrionic PD |  |  | - * |  |  |  |  |  |
| Narcissistic PD |  |  | - * |  |  |  |  |  |
| Cluster C PDs |  |  |  |  |  |  |  |  |
| Avoidant PD |  |  |  |  |  |  |  |  |
| Dependent PD |  |  | - * |  |  |  |  |  |
| Obsessive-compulsive PD |  |  |  |  |  |  |  |  |
| *<55 years* |  |  |  |  |  |  |  |  |

*= non-significant association; †= Analyses derived from the same data source; different confounders were assessed in the fully adjusted models

**Supplementary Table 8:** Evidence gap analysis of associations between arthritis (exposure) and PDs (outcome)

| **Type of PD/grouping** | **No evidence** | **Inconsistent Evidence** | **Limited Evidence** | | | | **Moderate Evidence** | **Strong Evidence** |
| --- | --- | --- | --- | --- | --- | --- | --- | --- |
|  |  | *If ≤75% studies reported consistent findings* |  | *Generally consistent findings in:* | | | *Generally consistent findings in:* | *Generally consistent findings in:* |
|  |  |  | 1 high-quality cross-sectional study | 2 high-quality cross-sectional studies | ≥ 3 high-quality cross-sectional studies | 1 or 2 high-quality case-control studies | 1 high-quality cohort study and ≥2 high quality case-control studies | Multiple high-quality cohort studies |
| Any PD |  |  |  |  |  |  |  |  |
| Probable PD |  |  |  |  |  |  |  |  |
| Cluster A PDs |  |  |  |  |  |  |  |  |
| Paranoid PD |  |  |  |  |  |  |  |  |
| Schizoid PD |  |  |  |  |  |  |  |  |
| Schizotypal PD |  |  |  |  |  |  |  |  |
| Cluster B PDs |  |  |  |  |  |  |  |  |
| Antisocial PD |  |  |  |  |  |  |  |  |
| Borderline PD |  |  |  |  |  |  |  |  |
| Borderline PD features/symptoms |  |  |  |  |  |  |  |  |
| Histrionic PD |  |  |  |  |  |  |  |  |
| Narcissistic PD |  |  |  |  |  |  |  |  |
| Cluster C PDs |  |  |  |  |  |  |  |  |
| Avoidant PD |  |  |  |  |  |  |  |  |
| Dependent PD |  |  | - * |  |  |  |  |  |
| Obsessive-compulsive PD |  |  |  |  |  |  |  |  |

*= non-significant association

**Supplementary Table 9:** Evidence gap analysis of associations between PDs (exposure) and back/neck/spinal pain (outcome)

| **Type of PD/grouping** | **No evidence** | **Inconsistent Evidence** | **Limited Evidence** | | | | **Moderate Evidence** | **Strong Evidence** |
| --- | --- | --- | --- | --- | --- | --- | --- | --- |
|  |  | *If ≤75% studies reported consistent findings* |  | *Generally consistent findings in:* | | | *Generally consistent findings in:* | *Generally consistent findings in:* |
|  |  |  | 1 high-quality cross-sectional study | 2 high-quality cross-sectional studies | ≥ 3 high-quality cross-sectional studies | 1 or 2 high-quality case-control studies | 1 high-quality cohort study and ≥2 high quality case-control studies | Multiple high-quality cohort studies |
| Any PD |  |  |  |  |  |  |  |  |
| Probable PD |  |  |  |  |  |  |  |  |
| Cluster A PDs |  |  |  |  |  |  |  |  |
| Paranoid PD |  |  |  |  |  |  |  |  |
| Schizoid PD |  |  |  |  |  |  |  |  |
| Schizotypal PD |  |  |  |  |  |  |  |  |
| Cluster B PDs |  |  |  |  |  |  |  |  |
| Antisocial PD |  |  |  |  |  |  |  |  |
| Borderline PD |  |  |  |  |  |  |  |  |
| Borderline PD features/symptoms |  |  |  |  |  |  |  |  |
| Histrionic PD |  |  |  |  |  |  |  |  |
| Narcissistic PD |  |  |  |  |  |  |  |  |
| Cluster C PDs |  |  |  |  |  |  |  |  |
| Avoidant PD |  |  |  |  |  |  |  |  |
| Dependent PD |  |  |  |  |  |  |  |  |
| Obsessive-compulsive PD |  |  |  |  |  |  |  |  |

Supplementary Table 10: Evidence gap analysis of associations between back/neck/spinal pain (exposure) and PDs (outcome)

| **Type of PD/grouping** | **No evidence** | **Inconsistent Evidence** | **Limited Evidence** | | | | **Moderate Evidence** | **Strong Evidence** |
| --- | --- | --- | --- | --- | --- | --- | --- | --- |
|  |  | *If ≤75% studies reported consistent findings* |  | *Generally consistent findings in:* | | | *Generally consistent findings in:* | *Generally consistent findings in:* |
|  |  |  | 1 high-quality cross-sectional study | 2 high-quality cross-sectional studies | ≥ 3 high-quality cross-sectional studies | 1 or 2 high-quality case-control studies | 1 high-quality cohort study and ≥2 high quality case-control studies | Multiple high-quality cohort studies |
| Any PD |  |  |  |  |  |  |  |  |
| Probable PD |  |  |  |  |  |  |  |  |
| Cluster A PDs |  |  |  |  |  |  |  |  |
| Paranoid PD |  |  |  |  |  |  |  |  |
| Schizoid PD |  |  |  |  |  |  |  |  |
| Schizotypal PD |  |  |  |  |  |  |  |  |
| Cluster B PDs |  |  |  |  |  |  |  |  |
| Antisocial PD |  |  |  |  |  |  |  |  |
| Borderline PD |  |  |  |  |  |  |  |  |
| Borderline PD features/symptoms |  |  |  |  |  |  |  |  |
| Histrionic PD |  |  |  |  |  |  |  |  |
| Narcissistic PD |  |  |  |  |  |  |  |  |
| Cluster C PDs |  |  |  |  |  |  |  |  |
| Avoidant PD |  |  |  |  |  |  |  |  |
| Dependent PD |  |  |  |  |  |  |  |  |
| Obsessive-compulsive PD |  |  |  |  |  |  |  |  |

**Supplementary Table 11:** Evidence gap analysis of associations between PDs (exposure) and fibromyalgia/muscular pain (outcome)

| **Type of PD/grouping** | **No evidence** | **Inconsistent Evidence** | **Limited Evidence** | | | | **Moderate Evidence** | **Strong Evidence** |
| --- | --- | --- | --- | --- | --- | --- | --- | --- |
|  |  | *If ≤75% studies reported consistent findings* |  | *Generally consistent findings in:* | | | *Generally consistent findings in:* | *Generally consistent findings in:* |
|  |  |  | 1 high-quality cross-sectional study | 2 high-quality cross-sectional studies | ≥ 3 high-quality cross-sectional studies | 1 or 2 high-quality case-control studies | 1 high-quality cohort study and ≥2 high quality case-control studies | Multiple high-quality cohort studies |
| Any PD |  |  |  |  |  |  |  |  |
| Cluster A PDs |  |  |  |  |  |  |  |  |
| Paranoid PD |  |  |  |  |  |  |  |  |
| Schizoid PD |  |  |  |  |  |  |  |  |
| Schizotypal PD |  |  |  |  |  |  |  |  |
| Cluster B PDs |  |  |  |  |  |  |  |  |
| Antisocial PD |  |  |  |  |  |  |  |  |
| Borderline PD |  |  |  |  |  |  |  |  |
| Borderline PD features/symptoms |  |  |  |  |  |  |  |  |
| Histrionic PD |  |  |  |  |  |  |  |  |
| Narcissistic PD |  |  |  |  |  |  |  |  |
| Cluster C PDs |  |  |  |  |  |  |  |  |
| Avoidant PD |  |  |  |  |  |  |  |  |
| Avoidant PD |  |  |  |  |  |  |  |  |
| Dependent PD |  |  |  |  |  |  |  |  |
| Obsessive-compulsive PD |  |  |  |  |  |  |  |  |

**Supplementary Table 12:** Evidence gap analysis of associations between fibromyalgia/muscular pain (exposure) and PDs (outcome)

| **Type of PD/grouping** | **No evidence** | **Inconsistent Evidence** | **Limited Evidence** | | | | **Moderate Evidence** | **Strong Evidence** |
| --- | --- | --- | --- | --- | --- | --- | --- | --- |
|  |  | *If ≤75% studies reported consistent findings* |  | *Generally consistent findings in:* | | | *Generally consistent findings in:* | *Generally consistent findings in:* |
|  |  |  | 1 high-quality cross-sectional study | 2 high-quality cross-sectional studies | ≥ 3 high-quality cross-sectional studies | 1 or 2 high-quality case-control studies | 1 high-quality cohort study and ≥2 high quality case-control studies | Multiple high-quality cohort studies |
| Any PD |  |  |  |  |  |  |  |  |
| Cluster A PDs |  |  |  |  |  |  |  |  |
| Paranoid PD |  |  |  |  |  |  |  |  |
| Schizoid PD |  |  |  |  |  |  |  |  |
| Schizotypal PD |  |  |  |  |  |  |  |  |
| Cluster B PDs |  |  |  |  |  |  |  |  |
| Antisocial PD |  |  |  |  |  |  |  |  |
| Borderline PD |  |  |  |  |  |  |  |  |
| Histrionic PD |  |  |  |  |  |  |  |  |
| Narcissistic PD |  |  |  |  |  |  |  |  |
| Cluster C PDs |  |  |  |  |  |  |  |  |
| Avoidant PD |  |  |  |  |  |  |  |  |
| Dependent PD |  |  |  |  |  |  |  |  |
| Obsessive-compulsive PD |  |  |  |  |  |  |  |  |

**Supplementary Table 13:** Evidence gap analysis of associations between PDs (exposure) and bone mineral density (outcome)

| **Type of PD/grouping** | **No evidence** | **Inconsistent Evidence** | **Limited Evidence** | | | | **Moderate Evidence** | **Strong Evidence** |
| --- | --- | --- | --- | --- | --- | --- | --- | --- |
|  |  | *If ≤75% studies reported consistent findings* |  | *Generally consistent findings in:* | | | *Generally consistent findings in:* | *Generally consistent findings in:* |
|  |  |  | 1 high-quality cross-sectional study | 2 high-quality cross-sectional studies | ≥ 3 high-quality cross-sectional studies | 1 or 2 high-quality case-control studies | 1 high-quality cohort study and ≥2 high quality case-control studies | Multiple high-quality cohort studies |
| Any PD |  |  | - * |  |  |  |  |  |
| Cluster A PDs |  |  |  |  |  |  |  |  |
| Paranoid PD |  |  |  |  |  |  |  |  |
| Schizoid PD |  |  |  |  |  |  |  |  |
| Schizotypal PD |  |  |  |  |  |  |  |  |
| Cluster B PDs |  |  | - * |  |  |  |  |  |
| Antisocial PD |  |  |  |  |  |  |  |  |
| Borderline PD |  |  |  |  |  |  |  |  |
| Borderline PD features/symptoms |  |  |  |  |  |  |  |  |
| Histrionic PD |  |  |  |  |  |  |  |  |
| Narcissistic PD |  |  |  |  |  |  |  |  |
| Cluster C PDs |  |  | - * |  |  |  |  |  |
| Avoidant PD |  |  |  |  |  |  |  |  |
| Dependent PD |  |  |  |  |  |  |  |  |
| Obsessive-compulsive PD |  |  |  |  |  |  |  |  |

*= non-significant association

**Supplementary Table 14:** Evidence gap analysis of associations between bone mineral density (exposure) PDs (outcome)

| **Type of PD/grouping** | **No evidence** | **Inconsistent Evidence** | **Limited Evidence** | | | | **Moderate Evidence** | **Strong Evidence** |
| --- | --- | --- | --- | --- | --- | --- | --- | --- |
|  |  | *If ≤75% studies reported consistent findings* |  | *Generally consistent findings in:* | | | *Generally consistent findings in:* | *Generally consistent findings in:* |
|  |  |  | 1 high-quality cross-sectional study | 2 high-quality cross-sectional studies | ≥ 3 high-quality cross-sectional studies | 1 or 2 high-quality case-control studies | 1 high-quality cohort study and ≥2 high quality case-control studies | Multiple high-quality cohort studies |
| Any PD |  |  |  |  |  |  |  |  |
| Probable PD/personality problems |  |  |  |  |  |  |  |  |
| Cluster A PDs |  |  |  |  |  |  |  |  |
| Paranoid PD |  |  |  |  |  |  |  |  |
| Schizoid PD |  |  |  |  |  |  |  |  |
| Schizotypal PD |  |  |  |  |  |  |  |  |
| Cluster B PDs |  |  |  |  |  |  |  |  |
| Antisocial PD |  |  |  |  |  |  |  |  |
| Borderline PD |  |  |  |  |  |  |  |  |
| Borderline PD features/symptoms |  |  |  |  |  |  |  |  |
| Histrionic PD |  |  |  |  |  |  |  |  |
| Narcissistic PD |  |  |  |  |  |  |  |  |
| Cluster C PDs |  |  |  |  |  |  |  |  |
| Avoidant PD |  |  |  |  |  |  |  |  |
| Dependent PD |  |  |  |  |  |  |  |  |
| Obsessive-compulsive PD |  |  |  |  |  |  |  |  |
